# Integrative effect of yoga practice in patients with knee arthritis: A PRISMA-compliant meta-analysis

**DOI:** 10.1097/MD.0000000000011742

**Published:** 2018-08-03

**Authors:** Yiguo Wang, Shibi Lu, Ruomei Wang, Peng Jiang, Feng Rao, Bo Wang, Yong Zhu, Yihe Hu, Jianxi Zhu

**Affiliations:** a School of Medicine, Nankai University, Tianjin; b Beijing Key Lab of Regenerative Medicine in Orthopedics, Key Laboratory of Musculoskeletal Trauma & War Injuries, PLA Institute of Orthopedics, Chinese PLA General Hospital, Beijing; c Department of Endocrinology, The Fourth Affiliated Hospital of Anhui Medical University. Hefei; d Department of Trauma and Orthopedics, Peking University People's Hospital, 11th Xizhimen South Street, Beijing; e Department of Orthopaedics, Xiangya Hospital, Central South University, Changsha, Hunan, China.

**Keywords:** knee arthritis, meta-analysis, SF-36, WOMAC, yoga

## Abstract

**Background:**

Benefits of yoga practice in patients with knee osteoarthritis and rheumatoid arthritis remains controversial. This study performs a meta-analysis to quantify the efficiency of yoga exercise for patients pain reduction, functional recovery, and general wellbeing.

**Methods:**

A computerized search of PubMed and Embase was performed to identify relevant studies. The outcome measures were pain, stiffness, and physical function. Two investigators identified eligible studies and extracted data independently. The quality of citations was measured using Jadad score. Standardized mean differences (SMDs) with 95% confidence intervals (CIs) were calculated for pain, musculoskeletal impairment, quality of life, general wellbeing, and mental wellbeing.

**Results:**

A total of 13 clinical trials involving 1557 patients with knee osteoarthritis and rheumatoid arthritis were included in final meta-analysis with the average Jadad score 2.8. The SMD was −0.98 (95% CI −1.18, −0.78, *P < *.05) for pain, −1.83 (95% CI −2.09, −1.57, *P < *.05) for functional disability, was 0.80 (95% CI 0.59, 1.01, *P < *.05) for Short Form 36 Health Survey (SF-36) general health, 0.49 (95% CI 0.14, 0.82, *P < *.05) for SF-36 mental health, and HAQ was −0.55 (95% CI −0.83, −0.26, *P < *.05) for health associated questionnaire (HAQ). All the results favor yoga training group.

**Conclusions:**

Regular yoga training is helpful in reducing knee arthritic symptoms, promoting physical function, and general wellbeing in arthritic patients.

## 1 Introduction

Arthritis is a common disease in senior associated with pain and dysfunction. Osteoarthritis (OA) and rheumatoid arthritis (RA) are 2 major forms of arthritis and they share high degree of similarity in symptoms and treatments. It is estimated that more than 21% of the adults or 46.4 million people suffered from arthritis^[1,2]^ in the United States. Furthermore, the economic burden to arthritic patients in the United States was up to $128 billion, equal to 1.2% of the US total annual income.^[3]^

Three major modalities of treatment applying for management of arthritic patients are drugs, nonpharmacological interventions, and surgical interventions.^[4]^ American College of Rheumatology and the Osteoarthritis Research Society International recommend (ACRORSI) recommends both pharmacologic and nonpharmacologic interventions as conservative treatments for arthritic patients. Current pharmacological treatments available of OA and RA majorly include nonsteroidal anti-inflammatory drugs (NSAIDs) and disease modifying osteoarthritic drugs (DMOADs).^[4]^ Meanwhile, the nonpharmacological treatments including exercise, weight loss, education, and physical therapy are also found to be effective for arthritic patients with less side effect and economic burden. Centers for Disease Control and Prevention and the Arthritis Foundation recommends exercise programs for OA should include flexibility, strengthening, endurance, and balance components.^[5]^ Several studies have designed a variety of physical exercises, including weightbearing, nonweightbearing, and neuromuscular strengthening to alleviate arthritic conditions. While there exist a plenty of physical exercises for knee OA, the best suitable type or dosage of exercise for knee arthritis remains uncertain.^[6]^

Yoga is a psycho-physical exercise with slow movements associated with muscle strengthening to improve one's physical and mental conditions.^[7]^ This ancient system of physical and mental exercise originated from Indus Valley civilization can be traced back to 200 BC. Contemporarily, yoga is described as “a systematic practice and implementation of mind and body in the living process of human beings to keep harmony within self, within society, and with nature.”^[8]^ In modern medical view, effects of yoga could influence people's musculoskeletal system, immune system, nervous system and sympathetic activity, and so on.^[9]^ Specifically, yoga exercise increases flexibility and muscle strength, physical balancing, improves fitness, and pain relieving.^[9]^ Its whole body benefits involve reducing distress, lowering blood pressure, and metabolic regulation. Comprising balancing, breathing and relaxation components, yoga as an alternative management for knee arthritis is becoming increasingly popular. Publications in patients with OA have shown inconsistency for pain, and physical function and spiritual relaxations after yoga exercise.^[10–15]^ To the best of our knowledge, there is no meta-analysis evaluating comprehensive effect size of yoga on knee arthritis pain relieving, restoration of joint function, and life quality.

Therefore, the aim of this research is to determine the comprehensive effectiveness of yoga on pain, functional, psychosocial, and life quality outcomes in people with clinically diagnosed knee osteoarthritis and rheumatoid arthritis. We performed a meta-analysis of existing literature to quantify the effects of yoga exercise on patients’ visual analog scale (VAS), Western Ontario and McMaster Universities Arthritis Index (WOMAC), the Short Form 36 Health Survey (SF-36), and Health Assessment Questionnaire (HAQ) in patients with OA. Findings and explanations of this meta-analysis will help provide a better understanding of yoga exercise in patients with knee arthritis and a reference to healthcare guidance.

## 2 Methods

The present meta-analysis was planned and conducted in accordance with Preferred Reporting Items for Systematic Reviews and Meta-Analyses (PRISMA) guidelines.^[16]^ The protocol was not registered on any database. Since all of the data were gained through open access literature on the Internet and no direct information was collected from patients, no ethical approval or statement of consent was needed in this study.

### 2.1 Literature search

An internet-based search was performed in 4 databases (PubMed/MEDLINE, Embase databases, Scopus, and Cochrane Library) using the combination of logic keywords related to yoga and knee arthritis. The reference of retrieved articles and reviews were identified to search related literature. In addition, unpublished and ongoing studies were searched manually using the following websites: ClinicalTrials.gov (http://www.clinicaltrials.gov/) and the World Health Organization International Clinical Trials Registry (http://apps.who.int/trialsearch/Default.aspx). The most recent internet-based search was performed on December 15, 2016 and no any language restriction was preimposed. All the keywords were constructed and with adaptation for each database if necessary. The complete searching strategy for PubMed/MEDLINE was as follows:

1. Yoga[MeSH Terms] OR Yoga∗[Title/Abstract] OR Yogic[Title/Abstract] OR Pranayam∗[Title/Abstract] OR Asana∗[Title/Abstract]
2. Osteoarthritis[MeSH Terms] OR osteoarthriti∗[Title/Abstract] OR osteoarthro$∗[Title/Abstract] OR osteo?arthritic$∗[Title/Abstract] OR gonarthriti$∗[Title/Abstract] OR gonarthro$∗[Title/Abstract] OR coxarthro$∗[Title/Abstract] OR arthros$∗[Title/Abstract] OR coxarthriti$∗[Title/Abstract] OR arthritis, rheumatoid“[MeSH Terms] OR(arthritis”[All Fields] AND “rheumatoid”[All Fields])
3. 1 AND 2
4. Randomized Controlled Trial[Publication Type] OR controlled clinical trial[Publication Type] OR randomized[Title/Abstract] OR random[Title/Abstract] OR placebo[Title/Abstract] OR randomly[Title/Abstract] OR trial[Title/Abstract] OR group[Title/Abstract]
5. 3 AND 4

### 2.2 Study selection

All the retrieved article abstracts and full texts were scrutinized by 2 independent viewers (YW and RW). All the extracted data were typed into an Excel file and reviewed by a third researcher (RF). If any contradiction was raised, a third person (SL) was required to discuss and reach an agreement. The articles included in the analysis should meet following criteria: knee OA or RA according to ACRORSI diagnostic criteria; yoga was used as an intervention without other treatment; studies including VAS, WOMAC, SF-36, HAQ used as outcome parameters; study design, controlled trial, cohort study; and availability of full text. Those trials whose additional parameters (e.g., education or other type of exercise) was unbalanced between the experimental group and the control group were ruled out.^[4,13]^

### 2.3 Quality assessment

Two researchers (YW and RW) independently evaluated the methodological quality of the included citations. The Jadad score^[17]^ was utilized to evaluate the methodological quality of each clinical trial. A score ranged from 0 to 5 according to the descriptions of randomization (0–2 points), blinding method (0–2 points) and reporting of participant withdrawals (0–1 point). The quality scale ranges from 0 to 5 points with a higher score indicating better quality of paper. For the study quality assessment in final, if the Jadad score was 2, it was treated as low-quality citation and higher than 3 as a high quality citation.

### 2.4 Data analysis

The meta-analysis program of the Cochrane Collaboration (Review Manager 5.3 and STATA 13.0) was employed for data collection and quantitative analysis. Since all the measures were continuous parameters, the mean difference was calculated using the standard mean difference (SMD). Random effects model was used to assess the SMDs with the associated 95% confidence intervals (CIs) from every study.^[22]^ Statistical heterogeneity between each citation was determined and quantified by the *I*^2^ parameter. The amplitude of study heterogeneity was categorized as low (*I*^2^ = 0%–24%), moderate (*I*^2^ = 25%–49%), substantial (*I*^2^ = 50%–74%), and considerable (*I*^2^ = 75%–100%).^[18]^ Summary estimates of the overall effect of treatment are provided in the form of a forest plot. The Mantel–Haenszel (M-H) method was utilized to synthesize combined result using fixed or random effect models depending on sample heterogeneity. A potential publication bias was assessed by generating Begg's funnel plot and Egger's regression plot.^[19]^ For all statistical analysis, *P < *.05 was considered to be statistically significant.

## 3 Results

### 3.1 Search results

**Figure 1:**
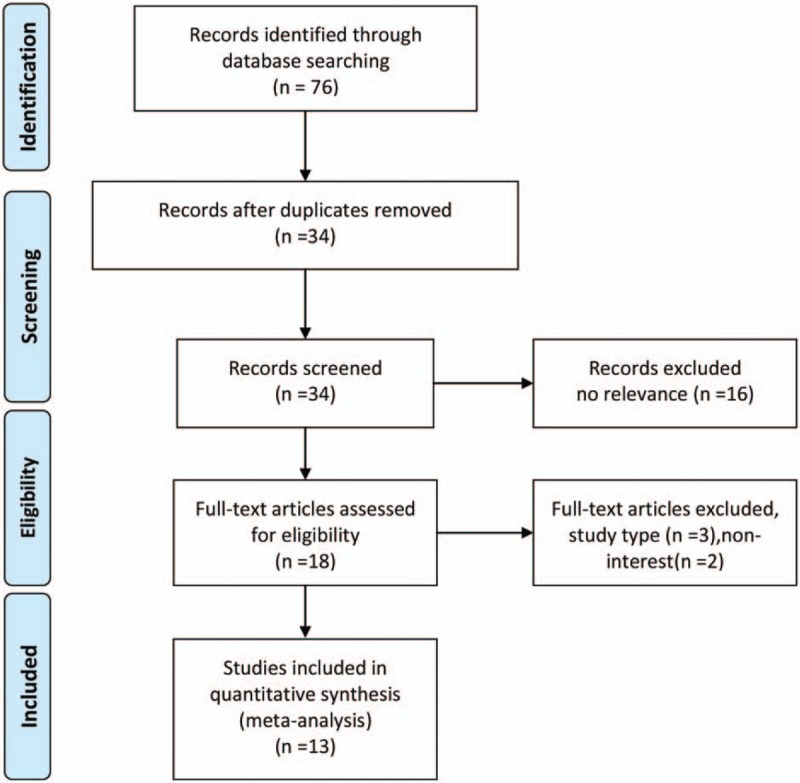
PRISMA flow chart of study selection. PRISMA = Preferred Reporting Items for Systematic Reviews and Meta-Analyses.

After initial internet-based search 76 relevant citations were browsed, of which 33 citations were ruled out because of duplication, no relevance or research type (review, no control, etc.) by viewing the titles and abstracts (Fig. 1). Around 34 potentially relevant studies were identified for full-text analysis, but 3 RCTs were excluded because of designing type (protocol articles) and 2 RCTs were excluded because it studied subjects with arthritis outside knee joint. Finally, 13 trials were selected for this meta-analysis and all of them were published in English language.

**Figure 2:**
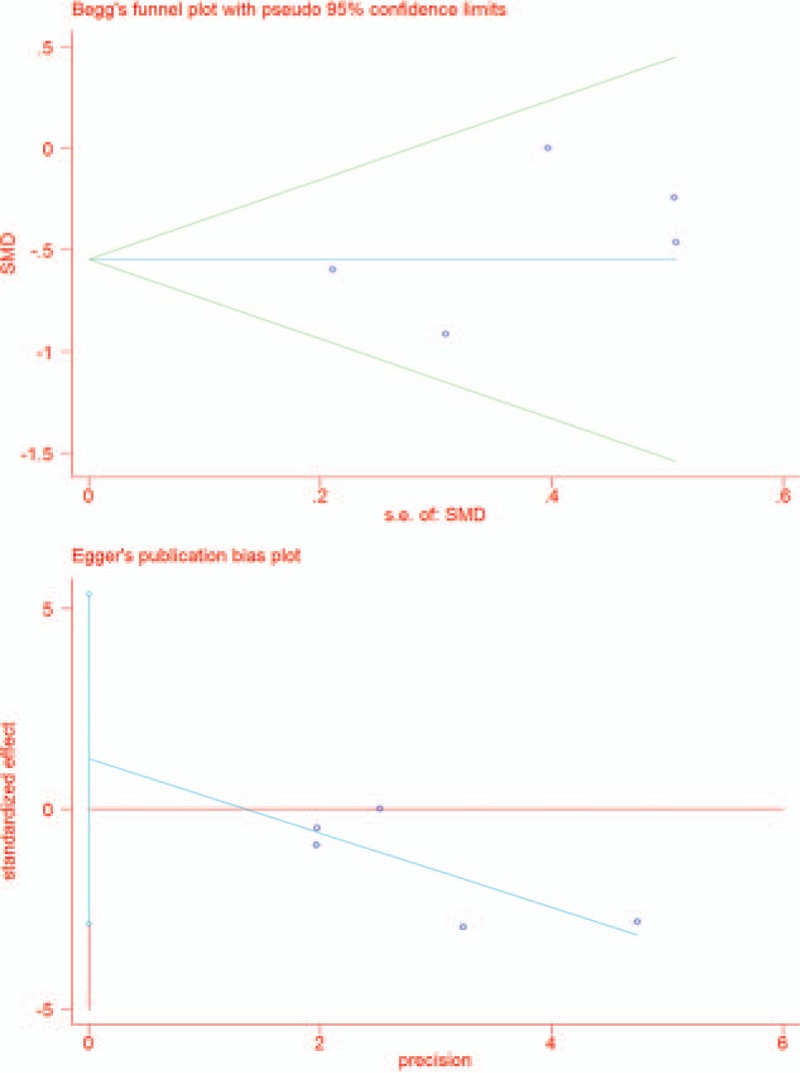
Begg's funnel plot and Egger's publication bias analysis.

The basic characteristics of the included citations were presented in Table 1. The studies date ranged from 2005 to 2015 with a total number of 1557 participants. Of the included papers, 7 researches selected knee OA patient as subject and 5 selected RA, and 1 select both OA, and RA patients. In yoga intervention, 8 studies chose Hata yoga, 4 chose Iyengar, and 1chose Raj yoga as experimental group. The exercise duration ranged from 45 minutes to 2.5 hours and training period lasted from 1 week to 12 weeks. For quality control, 2 investigators (SWP and LYS) independently evaluated Jadad score of every included article. The mean Jadad score for the studies was 2.8. For each main outcome of interest respective funnel plots were generated for evaluation of publication bias. Begg's funnel plot did not show any substantial asymmetry (Fig. 2). Egger's regression test (Fig. 2) indicated little evidence of publication bias (*P* > .05).

**Table 1:**
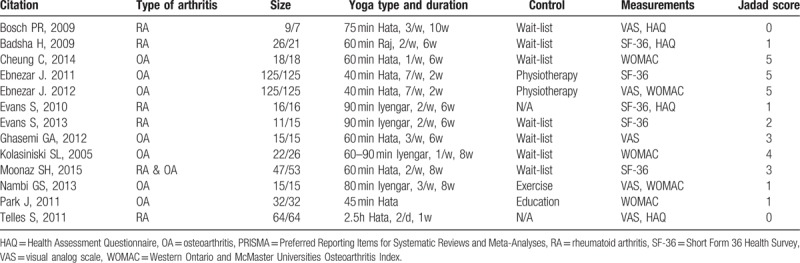
Basic characteristics of included citations.

### 3.2 Functional outcomes

**Figure 3:**
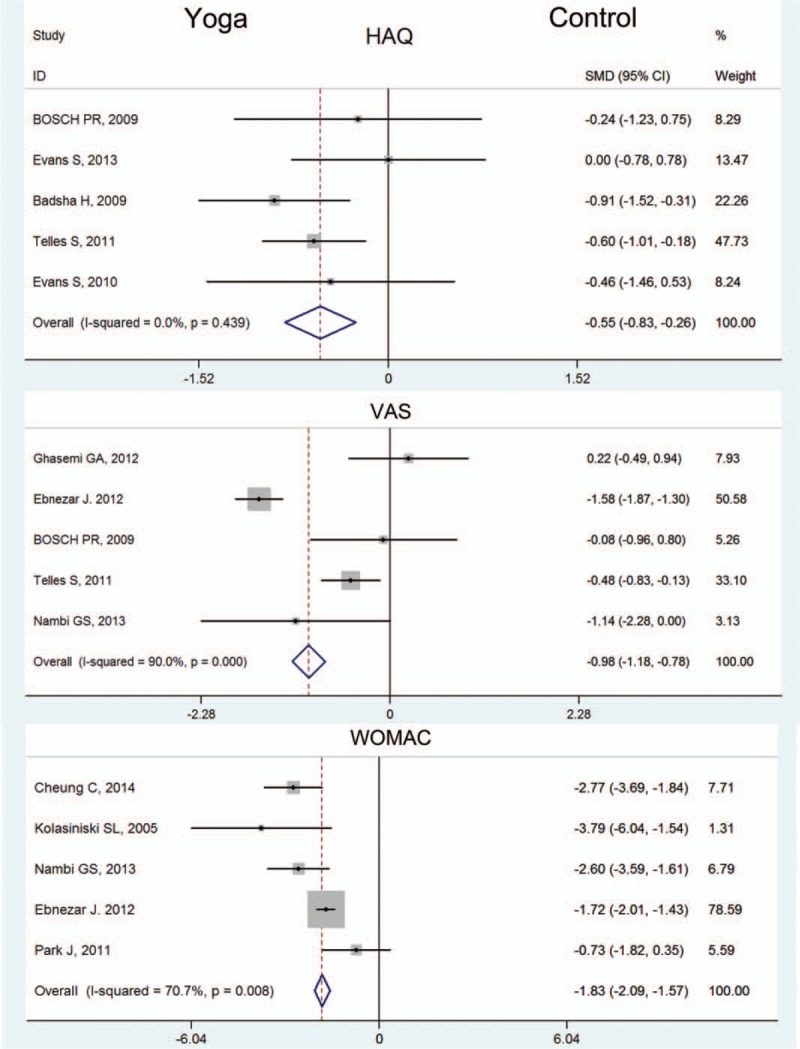
Forest plot of HAQ, VAS, and WOMAC. HAQ = Health Assessment Questionnaire, VAS = visual analog scale, WOMAC = Western Ontario and McMaster Universities Osteoarthritis Index.

**Figure 4:**
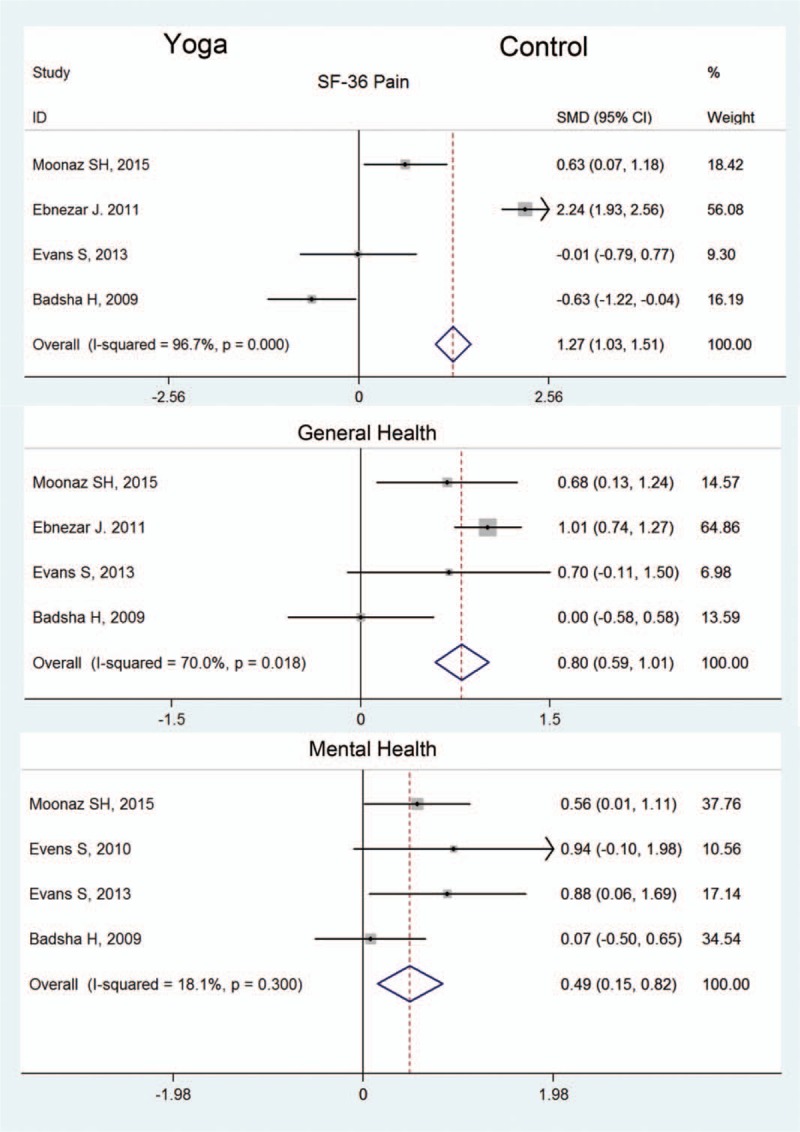
Forest plot of SF-36 pain, general health, and mental health. SF-36 = Short Form 36 Health Survey.

Parameters for pain measurement recruited in our analysis included VAS and SF-36 pain. Meta-analysis of 5 trials^[11,13]^ with 454 participants provided evidence that yoga effectively lowered knee VAS compared with control groups (Fig. 3). The SMD of VAS was −0.98 (95% CI −1.18, −0.78, *P < *.05, *P* for heterogeneity <.05, *I*^2^ = 90.0%) that favored yoga group. At the same time, 4 citations^[10,15,20]^ with a total of 423 participants referred SF-36 pain (Fig. 4). The SMD of SF-36 pain was 1.27 (95% CI 1.03, 1.51, *P < *.05, *P* for heterogeneity <.05, *I*^2^ = 96.7%) which favored yoga group. The synthetic results of these researches suggested that yoga training was beneficial in pain reduction.

Meta-analysis of 5 trials with 428 participants showed evidence for improved knee function after yoga training period (Fig. 3). The SMD of WOMAC was −1.83 (95% CI −2.09, −1.57, *P < *.05, *P* for heterogeneity <.05, *I*^2^ = 70.7%) that favored yoga group. The pooled analysis indicated that yoga training was superior than control in restoring knee functions.

### 3.3 Health outcomes

The outcome of SF-36 and HAQ provided comprehensive evidence for physical health and psychosocial changes in patients during yoga training period. The SF-36 mainly evaluated patients’ general health and mental health during experiment.

4 citations with 423 participants reported SF-36 general health (Fig. 3). The SMD of SF-36 general health was 0.80 (95% CI 0.59, 1.01, *P < *.05, *P* for heterogeneity <.05, *I*^2^ = 70%) that favored yoga group. This meta-analysis showed yoga training was beneficial for patients’ general health conditions compared with control group. Meanwhile, 4 citations^[10,15,20]^ with 205 participants reported SF-36 mental health score (Fig. 4). The SMD of SF-36 mental health was 0.49 (95% CI 0.14, 0.82, *P < *.05, *P* for heterogeneity >.05, *I*^2^ = 18.1%) that also favored yoga group. This meta-analysis showed yoga training was also effective in improving patients’ mental health conditions compared with control group.

HAQ was also a comprehensive method used to assess patients’ health related ability in arthritis.^[21]^ In our research, 5 citations^[10,20]^ with a total of 249 participants was involved in meta-analysis (Fig. 4). The SMD of HAQ was −0.55 (95% CI −0.83, −0.26, *P < *.05, *P* for heterogeneity >.05, *I*^2^ = 0%) that also favored yoga group. This result suggested yoga training was significant in improving patients’ health related conditions.

## 4 Discussion

Proper form and intensity of physical exercise can benefit patients with knee arthritis.^[4,22–24]^ This PRISMA style meta-analysis shows evidence that yoga practice can have comprehensive effect on patients with knee arthritis by pain reduction, improvement of joint function and life quality. A total of 13 citations assessing the effect of yoga in various knee arthritic conditions were screened through a search of 4 databases.

Pain reduction and function improvement are 2 primary outcomes in evaluating yoga efficiency in knee arthritis. A period of yoga practice interventions show result in clinically significant pain alleviation and functional improvements compared with control group. Of all knee OA and RA symptoms, regular yoga training seems to best alleviate pain sensation. The VAS and SF-36 pain scores in all the included citations were significantly lower in yoga group than in control group. Although the exact mechanism how yoga practice help reduce pain is not well understood, observations were made to the underlying actions of yoga for acute pain sensation, chronic pain sensitization and central nervous system sensitizations. Firstly, yoga provides the local structure strengthening to reduce physical pain by increasing joint stability. Knee muscle power reinforcement is one of the primary goals of OA practice since muscular weakness is the leading cause of OA pain and disability.^[25]^ Secondly, right way of body position gives rest and reduction of stress in specific area during yoga practicing. And this stress reducing effect also seems to be an effective mechanism in pain management in knee arthritis patients.^[26]^ Then, systematic effect like lowered heart rate and increased respiratory volume and other body responses on to stress may work synergistically to alleviate pain by regulating sympathetic and parasympathetic tone. As a result, patients knee function can be improved by reduction of pain, gain of knee joint stability and flexibility. Besides knee OA and RA, regular yoga practice also has a similar pain reducing effect in patients with low back pain,^[27]^ carpal tunnel syndrome,^[28]^ and hand painfulness^[29]^ et al.

Besides pain and functional recovery, regular yoga practice also provides people with multifactorial approach toward general wellbeing. Besides major local practices of the knee (asanas),it also bring benefits trainee at systematic level including respiratory promotion (pranayama), meditation (dharana), and stress alleviation(jnana).^[13]^ These systematic effects also help patient's pain reduction and functional improvements. Our meta-analysis showed that various forms of yoga training can increase patients’ mental health conditions. This good mental condition may be associated with attenuation in arthritic pain. Since pain sensation is a combination of peripheral mechanism and subjective, it can be influenced by the way a person sees the world and attributes meaning to the events. Yoga promotes the concepts of active mental awareness. Yoga practitioners were found to be more accepting of their condition and better able to detach from their psychological experience of pain.

For the statistical analysis, we utilized Begg's funnel plot and Egger's plot to evaluate selective and publication bias for the pooled results. The outcome showed balanced distribution in the plots indicating a controlled size of bias in this study. However, for some of the parameters, certain level of heterogeneity still existed. These heterogeneities originated in 3 aspects: clinical diversity from the clinical aspect of diseases, methodological diversity derived from the differences in trial design, risk bias, such as randomization, blindness, sample size et al. Statistical diversity that include systemic errors and the existence of various biases. To the best of our knowledge, heterogeneity of this study majorly comes from clinical aspect like VAS and other emotional indexes. Fortunately, for some of the parameters like VAS, has an effect size favoring treatment groups even with heterogeneity. At the same time, future homogeneous studies with larger sample size are required to update this pool to reduce heterogeneity.

The limitations of this meta-analysis are acknowledged. Firstly, method of search strategy is somewhat restricted. We only include peer-reviewed original articles in this meta-analysis and, therefore a publication bias may occur. Language bias is also possible since we do not cover all languages other than English. Although the search strategy was comprehensive, only 2 articles were found by citation tracking method. It is true that OA and RA share a high degree of similarity concerning patient's symptoms and treatments, but pathogenesis of these 2 diseases are distinctive. Some kind of heterogeneities may be brought into analysis by combining OA and RA studies. Secondly, sample size of this meta-analysis is still limited. Although total sample size is 1557, only 5 of the 13 trials have more than50 participants in total. It is unavoidable to be subjective to justify trials with sufficient homogeneity into pooled analysis. A further limitation is that the quality of clinical trials is heterogeneous. Almost have of the citations are with a Jadad score≥3, but some of the citations are still in poor quality (2 citations with Jadad score 0). This heterogeneity also reduces the power of the results.

Additional research is needed to evaluate yoga as an effective intervention for managing OA symptoms and improving function. Studies are needed that provide evidence of the most appropriate yoga exercise prescription (e.g., type, duration, intensity, and frequency) as well as to determine the optimal adherence rate to achieve satisfactory outcomes for people with OA. The practice of yoga may be an important complementary therapy to manage symptoms and benefit the overall health of persons with OA based on a holistic approach. This review indicates that yoga intervention could be used for relieving OA pain; however, in the absence of high-quality studies with low risk of bias, the true benefits of yoga, although promising, are still undetermined. Despite methodological limitations of the reviewed studies, yoga can be considered a safe complementary therapeutic option for individuals with OA.

## Author contributions

YW and YZ searched and evaluated citations, SL, RW, and JP conducted statistical analysis. YH, FR, and JZ wrote the main manuscript; JZ prepared all the figures. All authors reviewed the manuscript.

**Conceptualization:** Shibi Lu, Peng Jiang, Feng Rao, Bo Wang, Yong Zhu, Yihe Hu, Jianxi Zhu.

**Data curation:** Jianxi Zhu.

**Formal analysis:** Bo Wang, Jianxi Zhu.

**Funding acquisition:** Yihe Hu.

**Investigation:** Yiguo Wang.

**Project administration:** Ruomei Wang, Peng Jiang.

**Resources:** Ruomei Wang.

**Software:** Yiguo Wang, Ruomei Wang, Peng Jiang, Feng Rao, Jianxi Zhu.

**Validation:** Peng Jiang.

**Visualization:** Peng Jiang.

**Writing – original draft:** Feng Rao.
